# Folic Acid and Creatine as Therapeutic Approaches to Lower Blood Arsenic: A Randomized Controlled Trial

**DOI:** 10.1289/ehp.1409396

**Published:** 2015-05-15

**Authors:** Brandilyn A. Peters, Megan N. Hall, Xinhua Liu, Faruque Parvez, Tiffany R. Sanchez, Alexander van Geen, Jacob L. Mey, Abu B. Siddique, Hasan Shahriar, Mohammad Nasir Uddin, Tariqul Islam, Olgica Balac, Vesna Ilievski, Pam Factor-Litvak, Joseph H. Graziano, Mary V. Gamble

**Affiliations:** 1 Department of Environmental Health Sciences,; 2 Department of Epidemiology, and; 3 Department of Biostatistics, Mailman School of Public Health, Columbia University, New York, New York, USA; 4 Lamont-Doherty Earth Observatory, Columbia University, Palisades, New York, USA; 5 Columbia University Arsenic Project in Bangladesh, Dhaka, Bangladesh

## Abstract

**Background:**

The World Health Organization estimates that > 140 million people worldwide are exposed to arsenic (As)–contaminated drinking water. As undergoes biologic methylation, which facilitates renal As elimination. In folate-deficient individuals, this process is augmented by folic acid (FA) supplementation, thereby lowering blood As (bAs). Creatinine concentrations in urine are a robust predictor of As methylation patterns. Although the reasons for this are unclear, creatine synthesis is a major consumer of methyl donors, and this synthesis is down-regulated by dietary/supplemental creatine.

**Objectives:**

Our aim was to determine whether 400 or 800 μg FA and/or creatine supplementation lowers bAs in an As-exposed Bangladeshi population.

**Methods:**

We conducted a clinical trial in which 622 participants were randomized to receive 400 μg FA, 800 μg FA, 3 g creatine, 3 g creatine+400 μg FA, or placebo daily. All participants received an As-removal filter on enrollment, and were followed for 24 weeks. After the 12th week, half of the two FA groups were switched to placebo to evaluate post-treatment bAs patterns.

**Results:**

Linear models with repeated measures indicated that the decline in ln(bAs) from baseline in the 800-μg FA group exceeded that of the placebo group (weeks 1–12: β= –0.09, 95% CI: –0.18, –0.01; weeks 13–24: FA continued: β= –0.12, 95% CI: –0.24, –0.00; FA switched to placebo: β= –0.14, 95% CI: –0.26, –0.02). There was no rebound in bAs related to cessation of FA supplementation. Declines in bAs observed in the remaining treatment arms were not significantly different from those of the placebo group.

**Conclusions:**

In this mixed folate-deficient/replete study population, 12- and 24-week treatment with 800 μg (but not 400 μg) FA lowered bAs to a greater extent than placebo; this was sustained 12 weeks after FA cessation. In future studies, we will evaluate whether FA and/or creatine altered As methylation profiles.

**Citation:**

Peters BA, Hall MN, Liu X, Parvez F, Sanchez TR, van Geen A, Mey JL, Siddique AB, Shahriar H, Uddin MN, Islam T, Balac O, Ilievski V, Factor-Litvak P, Graziano JH, Gamble MV. 2015. Folic acid and creatine as therapeutic approaches to lower blood arsenic: a randomized controlled trial. Environ Health Perspect 123:1294–1301; http://dx.doi.org/10.1289/ehp.1409396

## Introduction

Chronic arsenic (As) exposure currently affects well over 140 million people in at least 70 countries worldwide. In Asia, at least 60 million people are at risk of chronic As exposure, of whom 35 million reside in Bangladesh (Kinniburgh and Smedley 2001). In a survey of 4,997 tube wells conducted in 2000 in our study region of Araihazar, Bangladesh, 72% of wells exceeded the World Health Organization maximum contaminant level for As of 10 μg/L (van Geen et al. 2002). A subsequent survey of the area 12 years later revealed that the number of wells had roughly doubled, with a similar proportion of contaminated wells (van Geen et al. 2014). Exposure to inorganic As (InAs) is associated with increased risk for cancers of the skin, lung, and bladder, as well as cardiovascular disease, respiratory illness, and neurologic deficits (National Research Council 2013).

Arsenic is metabolized through a series of reactions in which arsenite (As^III^) is first methylated to form monomethylarsonic acid (MMA^V^). MMA^V^ can be reduced to monomethylarsonous acid (MMA^III^), which subsequently undergoes a second methylation, generating dimethylarsinic acid (DMA^V^) (Challenger 1945). The methylation reactions are catalyzed by As methyltransferase (AS3MT), with *S*-adenosylmethionine (SAM) as the methyl donor (Lin et al. 2002). Arsenic methylation plays a critical role in the elimination of As from tissue stores; AS3MT knockout mice treated with arsenate retain a significantly greater body burden of As, and excrete less As in urine, than wild-type mice (Hughes et al. 2010). Individuals with a higher percentage of MMA^III+V^ in urine (%uMMA) and a lower percentage of DMA in urine (%uDMA) have increased odds of bladder cancer, lung cancer, skin cancer, and skin lesions (Steinmaus et al. 2010). These findings suggest that complete methylation of As to DMA may reduce risk for As-related disease outcomes.

**Figure 1:**
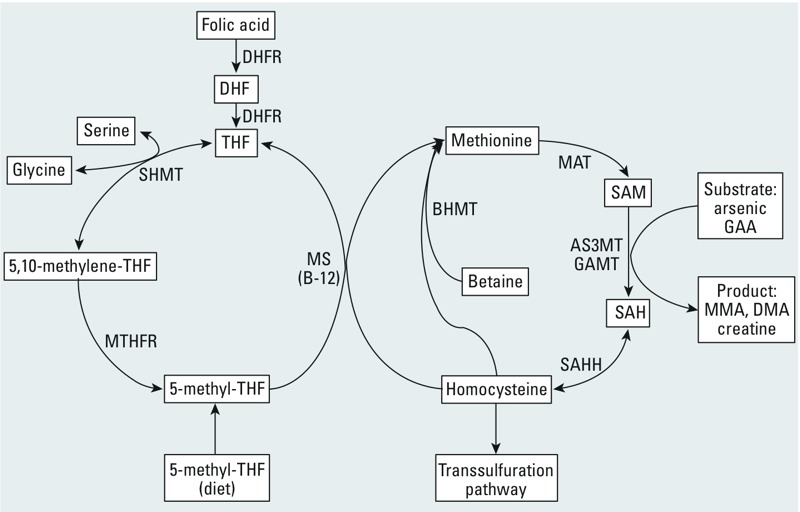
Overview of one-carbon metabolism. Folic acid (used in fortified foods and supplements) is reduced to dihydrofolate (DHF) and tetrahydrofolate (THF) by dihydrofolate reductase (DHFR). A methyl group is then transferred from serine to THF by serine hydroxymethyltransferase (SHMT), forming 5,10-methylene-THF and glycine. 5,10-methylene-THF can be reduced by 5,10-methylene tetrahydrofolate reductase (MTHFR) to 5-methyl-THF. The methyl group of 5-methyl-THF, the predominant naturally occurring form in dietary sources, is transferred to homocysteine by methionine synthase (MS), generating methionine and regenerating THF. Vitamin B_12_ is a cofactor for MS. Methionine can also be generated independently of folate and B_12_, by the action of betaine-homocysteine methyltransferase (BHMT), which transfers a methyl group from betaine to homocysteine. Methionine is then activated by methionine adenosyltransferase (MAT) to form *S*-adenosylmethionine (SAM), which serves as a universal methyl donor for numerous reactions, including the conversion of inorganic arsenic to methylated arsenicals (by arsenic methyltransferase; AS3MT), guanidinoacetate (GAA) to creatine (by guanidinoacetate methyltransferase; GAMT), and many others. The products of these methylation reactions are the methylated substrate and *S*-adenosylhomocysteine (SAH). SAH is subsequently hydrolyzed by SAH hydrolase (SAHH) to generate homocysteine. Homocysteine is then used either to regenerate methionine, or it is directed to the transsulfuration pathway.

SAM biosynthesis is regulated by one-carbon metabolism (Figure 1). This pathway is largely dependent on folate for recruitment of methyl groups; consequently, folate status is associated with individual variation in the ability to methylate As (Gamble et al. 2005b). Our previous survey of 1,650 adults in Bangladesh revealed a high prevalence of folate deficiency and hyperhomocysteinemia (Gamble et al. 2005a). In a 12-week randomized control trial (RCT), we demonstrated that supplementation of 400 μg/day folic acid (FA) to folate-deficient Bangladeshi adults significantly increased %uDMA, and lowered %uMMA and %uInAs as compared to placebo (Gamble et al. 2006). Moreover, FA supplementation resulted in a 14% reduction in blood As (bAs) and a 22% reduction in blood MMA (Gamble et al. 2007). In addition, in a nested case–control study we found folate deficiency to be associated with a 1.8 times higher odds [95% confidence interval (CI): 1.1, 2.9) of As-induced skin lesions (Pilsner et al. 2009). Collectively, these findings indicate that folate deficiency is a modifiable risk factor for As toxicity.

Our group (Ahsan et al. 2007; Gamble and Liu 2005; Gamble et al. 2005b, 2006; Hall et al. 2009) and others (Basu et al. 2011; Kile et al. 2009; Nermell et al. 2008) have previously reported that urinary creatinine (uCrn) is a strong predictor of As methylation capacity; it is positively associated with %uDMA, and negatively associated with %uInAs. The synthesis of creatine, the precursor of creatinine, consumes approximately 50% of all SAM-derived methyl groups (Stead et al. 2006). In omnivores, roughly half of creatine requirements are met through dietary intake of creatine, primarily from meat (Brosnan et al. 2011). UCrn is therefore a reflection of both dietary creatine intake and endogenous creatine synthesis (Levey et al. 1988). It is also commonly used in urinalyses to adjust for hydration status. Dietary creatine intake lowers creatine biosynthesis in rodents by inhibiting synthesis of guanidinoacetate (GAA), the precursor of creatine, thereby sparing methyl groups and lowering total homocysteine (tHcys) (Guthmiller et al. 1994; Stead et al. 2001). We hypothesized that dietary creatine intake may also facilitate the methylation of As, and may underlie the observed associations between the %uAs metabolites and uCrn.

In a new RCT, we sought to determine whether FA and/or creatine supplementation lowers bAs concentrations. Our primary objectives for this study were to determine whether *a*) 400-μg/day FA supplementation lowers bAs in the general population (both folate-deficient and -sufficient individuals), and whether a higher dose of FA (800 μg/day) provides an incremental benefit in lowering bAs; *b*) a rebound in bAs occurs after FA supplementation is ceased due to potential release of As from tissue stores; and *c*) bAs may also be lowered by reducing methylation demand (for endogenous creatine synthesis) via creatine supplementation.

## Methods

*The region and participants.* The Health Effects of Arsenic Longitudinal Study (HEALS) is a prospective cohort study that originally recruited 11,746 adults in 2000 living within a 25-km^2^ region in Araihazar, Bangladesh (Ahsan et al. 2006). The participants for the current study were recruited from the HEALS cohort. Eligible participants for the HEALS cohort were married adults 20–65 years of age who had been drinking from their current well for at least 3 years. For inclusion in the present study, the Folic Acid and Creatine Trial (FACT), participants were randomly selected from cohort participants who had been drinking from a household well having well water As > 50 μg/L for at least 1 year. Pregnant women, individuals taking nutritional supplements, individuals with protein in their urine, and individuals with known renal disease, diabetes, or gastrointestinal or other health problems were excluded from the study. Informed consent was obtained by our Bangladeshi field staff physicians. Ethical approval was obtained from the Institutional Review Board of Columbia University Medical Center and the Bangladesh Medical Research Council.

*Study design and fieldwork.* We recruited 622 participants who were randomized to one of five treatment groups: placebo (*n* = 104), 400 μg FA/day (FA400; *n* = 156), 800 μg FA/day (FA800; *n* = 154), 3 g creatine/day (*n* = 104), and 3 g creatine/day+FA400/day (*n* = 104) (FA and creatine supplements supplied by Douglas Laboratories, Pittsburgh, PA). The FA400 dose is based on the U.S. recommended dietary allowance (RDA) for adults, whereas the FA800 dose is twice the RDA (Institute of Medicine 1998). We based our creatine dose on the estimation of Brosnan et al. (2011) that the average creatine loss for a 20- to 39-year-old 70-kg male is 14.6 mmol/day (~ 1.9 g/day), with the average creatine losses for women 80% of that of men (Brosnan et al. 2011); we therefore concluded that 3 g creatine/day should be sufficient to downregulate endogenous creatine synthesis.

**Figure 2:**
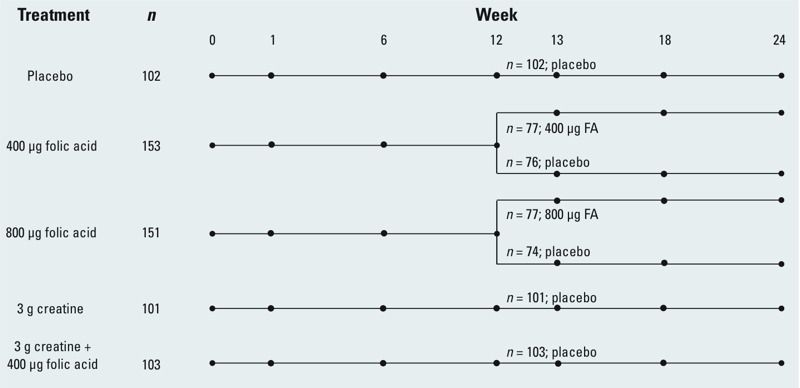
Overview of the FACT study design. At week 0 (baseline), all study participants were provided with arsenic removal water filters. Note changes in treatment at week 12. Dots (•) represent times of blood collection for the measurement of blood arsenic.

Randomization was performed separately for men and women and was conducted in blocks to ensure balance in the treatment groups at any specific time. A total of 12 participants were dropped over the course of the study for various reasons, including adverse events [*n* = 6; 1 in the placebo group (abdominal cramps), 1 in the FA400 group (hypertension), 3 in the FA800 group (abdominal cramps; severe vertigo; bilateral hydronephrosis), 1 in the creatine group (severe vertigo)], pregnancy (*n* = 3; FA400, creatine group, and creatine+FA400 group), missing baseline blood sample (*n* = 1; creatine group), and dropout (*n* = 2; placebo group and FA400 group). The final sample size (*n* = 610) by treatment group was as follows: placebo (*n* = 102), FA400 (*n* = 153), FA800 (*n* = 151), creatine (*n* = 101), and creatine+FA400 (*n* = 103). After week 12, the FA400 and FA800 groups were randomly divided so that half continued their assigned supplements [*n* = 77 in the FA400 group (FA400/FA), *n* = 77 in the FA800 group (FA800/FA)], and the other half received placebo [*n* = 76 in the FA400 group (FA400/placebo), *n* = 74 in the FA800 group (FA800/placebo)], for the remainder of the study until week 24 (Figure 2). Also, after week 12, participants in the creatine and creatine+FA400 groups were switched to placebo to maintain the study blind while terminating the intervention for these participants.

We refer to the first 12 weeks of the study as the “first phase” of the trial; the objective of the first phase was to determine treatment effects on bAs compared to placebo. We will refer to the period from week 12 to week 24 as the “second phase” of the trial; the objective of the second phase was to determine whether a rebound in bAs occurs after cessation of FA supplementation, compared with participants who continue taking FA supplements.

The primary outcome of the trial was bAs. Venous blood samples were collected at baseline, week 12, and week 24 for the measurement of bAs and other covariates [plasma folate, B_12_, homocysteine, creatine + creatinine (Cr+Crn), and GAA]. Additionally, finger-stick blood samples were collected at weeks 1, 6, 13, and 18 for measurement of bAs.

Five experienced teams, each having one interviewer and one physician (all specifically trained for this study) worked simultaneously to recruit and follow study participants through house-to-house visits. These teams were responsible for all visits in which blood and urine samples were collected. All participants received a household-level arsenic removal water filter (READ-F filter; Brota Services International, Bangladesh), one of six types approved by the Bangladesh government (Government of Bangladesh and United Nations 2010), at baseline for the provision of low-As water (< 10 μg/L), and were instructed to exclusively use filtered water for all drinking and cooking purposes.

Participants received and retained two bottles of pills at enrollment for the first phase: One bottle contained folate pills or folate-matched placebo pills, and the second bottle contained creatine or creatine-matched placebo pills. At week 12, participants received a third bottle of pills containing folate or folate-matched placebo pills for the second phase. Village health workers either witnessed or inquired about compliance on a daily basis. All bottles were retained and pill counts were conducted at the end of each phase of the study. Study investigators, fieldwork teams, village health workers, laboratory technicians, and study participants were blinded to study treatment for the entire duration of the study.

*Sample collection and handling.* Venous blood was collected into EDTA vacutainer tubes. We collected fingerstick blood at weeks 1, 6, 13, and 18. Participants’ fingers were punctured with sterile lancets, and the first droplet of blood was wiped away; approximately 500 μL of blood was then collected using BD Microtainer™ Plastic Capillary Blood Collectors. All samples were placed in IsoRack cool packs (Brinkmann Instruments) designed to maintain a sample temperature of 0°C for 6 hr; within 4 hr, samples were transported to our field clinic in Araihazar. Venous blood samples were centrifuged at 3,000 × *g* for 10 min at 4°C, and plasma was separated from cells. Aliquots of whole blood and plasma, and fingerstick whole blood samples were stored at –80°C and shipped on dry ice to Columbia University for analysis. Urine samples were collected in 50-mL acid-washed polypropylene tubes, kept in portable coolers, frozen at –20°C within 4 hr, and similarly shipped on dry ice.

*Water arsenic.* Nonfiltered water samples were tested at baseline to determine baseline As exposure. These water samples were collected in 20-mL polyethylene scintillation vials, and acidified to 1% with high-purity Optima HCl (Fisher Scientific) at least 48 hr before analysis (van Geen et al. 2007). Water samples were then analyzed by high-resolution inductively coupled plasma mass spectrometry after 1:10 dilution and addition of a germanium spike to correct for fluctuations in instrument sensitivity. The detection limit of the method is typically < 0.2 μg/L. Intra- and interassay coefficients of variation (CVs) for water As were 2% and 2.6%, respectively. Postfiltered water samples were tested in the field at baseline, week 12, and week 24 using Hach EZ Arsenic test kits to ensure As removal efficiency of filters. Approximately 45 filters were replaced fully or had repairs during the trial.

*Total urinary arsenic.* Total urinary As was measured by graphite furnace atomic absorption spectrophotometry using the AAnalyst 600 graphite furnace system (PerkinElmer) (Nixon et al. 1991). Intra- and interassay CVs for urinary As were 3.1% and 5.4%, respectively. We used a method based on the Jaffe reaction (Slot 1965) to measure uCrn concentrations. Intra- and interassay CVs for uCrn were 1.3% and 2.9%, respectively.

*Total blood arsenic.* As previously described (Hall et al. 2006), a PerkinElmer Elan DRC II ICP-MS equipped with an AS 93+ autosampler was used to analyze whole blood samples for total bAs concentrations. Intra- and interassay CVs for bAs were 2.7% and 5.7%, respectively.

*Folate and B12.* Plasma folate and B_12_ were measured by radioimmunoassay (SimulTRAC-SNB; MP Biomedicals). Folate was measured in whole blood hemolysate by radioimmunoassay (SimulTRAC-S; MP Biomedicals), and red blood cell (RBC) folate was calculated by dividing by [%Hematocrit/100]. Intra- and interassay CVs were 5% and 13% for plasma folate, 6% and 17% for plasma B_12_, and 4% and 9% for RBC folate, respectively. Plasma folate was measured for all treatment groups at baseline and 12 and 24 weeks; RBC folate was measured only in the placebo, FA400, and FA800 groups.

*Plasma total homocysteine.* Plasma tHcys concentrations were measured by high-performance liquid chromatography (HPLC) with fluorescence detection according to the method described by Pfeiffer et al. (1999). The intra- and interassay CVs for tHcys were 5% and 7%, respectively.

*Plasma Cr+Crn and GAA.* Plasma Cr+Crn and GAA were measured by HPLC with fluorescence detection according to the method described by Carducci et al. (2002). We used an Inertsil ODS-3 3-μm HPLC column 4.6 × 100 mm (GL Sciences Inc.) and an excitation and emission wavelengths of 335 and 435 nm, respectively. Intra- and interassay CVs for Cr+Crn were 7% and 9%, respectively, and for GAA were 8% and 9%, respectively. Plasma Cr+Crn and GAA were measured only in the placebo, creatine, and creatine+FA400 groups.

*Statistical analysis*. We calculated summary statistics to describe the sample characteristics. To detect treatment group differences in the baseline variables, we used the chi-square test and the Kruskal–Wallis test for categorical and continuous variables, respectively. Blood As, our primary outcome of interest, had a skewed distribution. We applied a natural logarithmic transformation to bAs (hereafter called ln-bAs) to stabilize its variance and to reduce the impact of extreme values in the parametric model–based analysis. We calculated the geometric mean (anti-log–transformed mean of ln-bAs) at each time point to describe the treatment group specific time trend. The mean within-person difference in ln-bAs between two time points indicates the within-person change over the time interval, and the anti-log transformed mean difference is the ratio of the geometric means at these two time points. We calculated geometric mean ratios and 95% CIs, by treatment group and time. We used the ratio of the geometric means at two specified time points (later/earlier) to determine the percent change in geometric mean of bAs over the time interval, using the equation: percent change = (geometric mean ratio – 1) × 100%. For example, a mean ratio of 0.90 implies a 10% decrease in the geometric mean, and a mean ratio of 1.10 implies a 10% increase in the geometric mean. Because venous bAs and fingerstick bAs were highly correlated in a pilot sample (*r* = 0.99, *n* = 8), we used the fitted model and our fingerstick bAs measures to calculate venous bAs from fingerstick bAs at weeks 1, 6, 13, and 18.

The five first phase treatment groups were placebo, FA400, FA800, creatine, or creatine+FA400. The second phase comparisons involved the five groups placebo/placebo, FA400/FA, FA400/placebo, FA800/FA, and FA800/placebo. We used two-sample *t*-tests to compare week-12 ln-bAs between the FA400/FA and FA400/placebo groups, and between the FA800/FA and FA800/placebo groups, in order to check for effective randomization at week 12. In order to examine rebound in bAs related to cessation of FA supplements, we compared the change in ln-bAs from week 12 to week 24 between the FA400/FA and FA400/placebo groups, and between the FA800/FA and FA800/placebo groups, using two-sample *t*-tests.

For our primary analysis, we modeled the treatment effect on bAs over the 24-week period using linear models with repeated-measures of ln-bAs. We included predictors for the pattern of time trend in placebo group, for the treatment groups, and for time by treatment interactions. The model parameters for the interaction terms indicate the treatment group differences in mean change in ln-bAs over time. Generalized estimation equations (GEE), which use all available data and account for within-subject correlations in the repeated measures, were used to estimate the regression parameters.

All analyses were performed as intent-to-treat. All statistical tests were two-sided with a significance level of 0.05. Analyses were conducted in R (version 3.1.0; R Core Team 2014) and SAS (version 9.3; SAS Institute Inc., Cary, NC).

## Results

*Characteristics and compliance.* Descriptive statistics for the sample characteristics are presented in Table 1. The treatment groups did not differ significantly at baseline on age, sex, history of smoking and betel nut use, body mass index, and sociodemographic indices such as education and land ownership. Additionally, the groups did not differ on nutritional variables including plasma folate, B_12_, tHcys, Cr+Crn, and GAA, nor on As exposure variables including water As, urinary As (micrograms per gram Cr), and bAs.

**Table 1:** Baseline characteristics of the participants in a folic acid and creatine randomized controlled trial.

Characteristic	Placebo (*n*= 102)	FA400 (μg) (*n *= 153)	FA800 (μg) (*n *= 151)	Creatine (*n *= 101)	Creatine+FA400 (μg) (*n *= 103)	*p*-Value^*a*^
Age (years)	38.0 ± 7.3^*b*^	39.0 ± 8.0	38.2 ± 8.1	38.3 ± 8.2	38.0 ± 7.7	0.85
Male (%)	50.0	50.3	49.7	50.5	50.5	0.99
Smoking (%)^*c*^	24.5	23.8	29.1	28.7	30.1	0.72
Betel nut use (%)^*c*^	28.4	23.8	24.5	24.8	20.4	0.77
Education (years)	3.5 ± 3.7	3.3 ± 3.6	3.5 ± 3.6	3.3 ± 3.6	3.9 ± 4.1	0.85
Owns land (%)^*d*^	46.1	50.3	48.3	47.5	43.1	0.85
Body mass index (kg/m^2^)^*e*^	20.4 ± 3.1	19.5 ± 2.3	19.8 ± 2.7	20.0 ± 3.0	19.5 ± 2.5	0.31
Red blood cell folate (nmol/L)	483.5 ± 189.0	498.4 ± 332.8	494.8 ± 172.8	NA^*f*^	NA^*f*^	0.66
Plasma folate (nmol/L)	16.6 ± 17.2	16.7 ± 14.2	17.9 ± 15.8	16.0 ± 7.9	15.4 ± 8.7	0.67
Percent folate deficient (< 9 nmol/L in plasma)	21.6	23.5	17.9	13.9	20.4	0.38
Plasma B_12_ (pmol/L)	225.0 ± 97.2	246.4 ± 130.7	248.3 ± 141.8	255.9 ± 141.0	236.9 ± 121.0	0.82
Percent B_12_ deficient (< 151 pmol/L)	24.5	24.2	25.8	19.8	24.3	0.87
Plasma homocysteine (μmol/L)^*g*^	13.9 ± 10.8	13.6 ± 8.8	13.6 ± 10.3	12.4 ± 5.5	12.8 ± 5.6	0.90
Percent hyperhomocysteinemia (≥ 13 μmol/L)^*g*^	42.2	36.6	39.3	38.6	37.9	0.93
Plasma creatine + creatinine (μmol/L)	81.3 ± 23.8	NA^*h*^	NA^*h*^	77.3 ± 23.0	81.3 ± 28.5	0.36
Plasma guanidinoacetate (μmol/L)	2.05 ± 0.66	NA^*h*^	NA^*h*^	1.95 ± 0.57	1.98 ± 0.67	0.57
Water As (μg/L)^*i*^	120.4 ± 80.2	126.6 ± 83.9	131.3 ± 141.6	146.6 ± 181.2	124.6 ± 76.6	0.93
Urinary As (μg/L)	137.8 ± 136.9	160.0 ± 163.7	145.3 ± 117.2	180.0 ± 211.6	177.9 ± 155.1	0.08
Urinary As (μg/g Cr)	303.5 ± 201.8	339.5 ± 325.0	307.4 ± 182.0	328.2 ± 252.6	312.1 ± 164.4	0.75
Blood As (μg/L)	9.7 ± 5.7	11.0 ± 9.8	10.0 ± 5.4	10.7 ± 8.2	10.5 ± 5.3	0.64
Urinary creatinine (mg/dL)	48.8 ± 35.1	57.7 ± 45.2	53.8 ± 41.3	57.3 ± 36.7	62.4 ± 47.2	0.26
NA, not available. Values are percent or mean ± SD. ^***a***^Kruskal–Wallis test was used for continuous variables, and chi-squared test for categorical variables. ^***b***^*X̄* ± SD (all such values). ^***c***^FA400 *n *= 151. ^***d***^Cr+FA *n *= 102. ^***e***^Placebo *n *= 101, FA400 *n *= 150, FA800 *n *= 148, Cr *n*= 98, Cr+FA *n *= 102. ^***f***^RBC folate only measured in placebo (*n *= 100), FA400 (*n *= 149), and FA800 (*n *= 148) groups. ^***g***^FA800 *n *= 150. ^***h***^Creatine+creatinine and guanidinoacetate only measured in placebo (*n *= 101), creatine (*n *= 101), and Cr+FA400 (*n *= 102) groups. ^***i***^Placebo *n *= 99, FA400 *n *= 152, Cr+FA *n *= 100.						

Pill counts were conducted at the end of the first phase and second phase of the trial, and compliance for each participant was calculated as the percentage of study pills taken. Compliance did not differ substantially between treatment groups or between the first phase and second phase of the trial (data not shown). Over the course of the trial, participants’ compliance ranged from 79.1% to 100.0%, and the median (interquartile range) compliance was 99.5% (98.3%, 100.0%).

**Figure 3:**
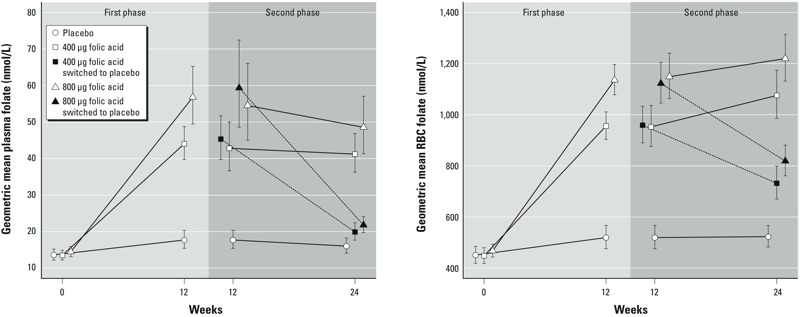
Plot of geometric mean (95% CI) plasma and red blood cell folate (nmol/L) by treatment group and time in the first phase and second phase of the trial. In the second phase, there are two values (white and black shapes) for each folic acid group: the white shapes represent the subset of participants in the folic acid groups that continued on folic acid, and the black shapes represent the subset of the participants in the folic acid groups that switched to placebo. Blood for measurement of folate was collected only at baseline, week 12, and week 24; intermediate time points are not available; therefore, the lines do not represent linear trends.

Plots of the time trends in geometric means of plasma folate and RBC folate, reflecting compliance with FA supplementation, are presented in Figure 3. As expected, plasma and RBC folate increased in both FA groups in the first phase of the trial, with a greater increase in the FA800 group than in the FA400 group. Additionally, participants who remained on FA supplementation in the second phase of the trial sustained the increase in plasma folate, whereas those who switched to placebo had a decrease in plasma folate in the second phase, returning close to the baseline concentration. RBC folate continued increasing during the second phase in participants who remained on FA, and decreased in those who switched to placebo, although not returning to baseline concentrations. The creatine group had a time trend of plasma folate similar to that of the placebo group, and the creatine+FA400 group had a time trend of plasma folate similar to the FA400 group (data not shown). The prevalence of folate deficiency (plasma folate < 9 nmol/L) decreased to < 1.5% at week 12 in groups receiving FA (compared with 17.9–23.5% at baseline), while not decreasing substantially in the placebo or creatine group (data not shown).

The within-person increases in plasma Cr+Crn (micromoles per liter) from baseline to week 12 in the creatine-treated groups (57.9 ± 80.1 in the creatine group, 51.6 ± 62.7 in the creatine+FA400 group, –2.1 ± 18.9 in the placebo group) also indicated high compliance with creatine supplementation. Additionally, both the creatine and creatine+FA400 groups experienced significant within-person declines in plasma GAA (micromoles per liter) from baseline to week 12, whereas the placebo group did not (–0.19 ± 0.54 in the creatine group, –0.17 ± 0.59 in the creatine+FA400 group, 0.06 ± 0.48 in the placebo group).

*Description of treatment effects in the first phase.* Table 2 presents geometric means of bAs and the % change in bAs from baseline to each follow-up time, along with 95% CIs, in the first phase of the study. After enrollment and following provision of an As removal filter, the geometric mean of bAs in the placebo group decreased through week 6, and then increased for the duration of the study (Tables 2 and 3). The other treatment arms had similar patterns in the geometric mean of bAs over time to that of the placebo group. A similar pattern was observed for urinary As (micrograms per gram creatinine) over time (see Supplemental Material, Table S1). FA800 was the only treatment group to attain lower geometric mean bAs than the placebo group at any follow-up time point (Tables 2 and 3). Additionally, the FA800 group had a greater percent decline in geometric mean bAs from baseline than the placebo group at the end of weeks 1, 6, and 12 (Table 2).

**Table 2:** Geometric mean (95% CI) of blood arsenic (μg/L) and the percent change in blood arsenic from baseline to week 12 by first phase treatment group.

Time or time comparison	Statistic	Placebo	FA400 (μg)	FA800 (μg)	Creatine	Creatine+FA400 (μg)
Baseline	Mean (95% CI)	8.32 (7.42, 9.32) (*n *= 102)	8.73 (7.89, 9.66) (*n *= 153)	8.72 (8.00, 9.50) (*n *= 151)	8.73 (7.72, 9.87) (*n *= 101)	9.26 (8.38, 10.24) (*n *= 103)
Week 1	Mean (95% CI)	6.40 (5.74, 7.14) (*n *= 102)	6.88 (6.18, 7.66) (*n *= 151)	6.20 (5.71, 6.74) (*n *= 151)	6.54 (5.83, 7.33) (*n *= 101)	6.84 (6.18, 7.58) (*n *= 102)
Week 6	Mean (95% CI)	6.20 (5.43, 7.09) (*n *= 102)	6.93 (6.17, 7.79) (*n *= 153)	5.86 (5.33, 6.43) (*n *= 151)	6.70 (5.92, 7.59) (*n *= 101)	7.09 (6.28, 8.01) (*n *= 103)
Week 12	Mean (95% CI)	7.62 (6.72, 8.64) (*n *= 101)	8.40 (7.55, 9.36) (*n *= 153)	7.19 (6.56, 7.87) (*n *= 150)	8.12 (7.20, 9.15) (*n *= 101)	7.96 (7.04, 9.00) (*n *= 103)
Week 1 vs. baseline	Percent change (95% CI)^*a*^	–23.0 (–26.6, –19.3) (*n *= 102)	–21.2 (–24.4, –17.8) (*n *= 151)	–28.9 (–31.8, –25.8) (*n *= 151)	–25.1 (–28.6, –21.4) (*n *= 101)	–25.6 (–29.7, –21.2) (*n *= 102)
Week 6 vs. baseline	Percent change (95% CI)	–25.4 (–31.5, –18.8) (*n *= 102)	–20.6 (–26.3, –14.5) (*n *= 153)	–32.8 (–38.0, –27.3) (*n *= 151)	–23.2 (–29.5, –16.3) (*n *= 101)	–23.5 (–30.8, –15.3) (*n *= 103)
Week 12 vs. baseline	Percent change (95% CI)	–9.5 (–16.5, –1.8) (*n *= 101)	–3.7 (–10.7, 3.8) (*n *= 153)	–17.8 (–25.0, –9.8) (*n *= 150)	–7.0 (–14.8, 1.5) (*n *= 101)	–14.0 (–22.2, –5.0) (*n *= 103)
^***a***^Percent change = (geometric mean ratio – 1) × 100.						

**Table 3:** Geometric mean (95% CI) of blood arsenic (μg/L) and the percent change in blood arsenic from week 12 to week 24 by second phase treatment group.

Time or time comparison	Statistic	Placebo	FA400 (μg) continued	FA400 (μg) switched to placebo	FA800 (μg) continued	FA800 (μg) switched to placebo
Week 12	Mean (95% CI)	7.62 (6.72, 8.64) (*n *= 101)	8.56 (7.33, 10.00) (*n *= 77)	8.25 (7.09, 9.60) (*n *= 76)	7.39 (6.56, 8.33) (*n *= 77)	6.97 (6.05, 8.04) (*n *= 73)
Week 13	Mean (95% CI)	7.37 (6.50, 8.35) (*n *= 102)	8.22 (6.93, 9.76) (*n *= 77)	8.28 (6.99, 9.82) (*n *= 75)	7.04 (6.21, 7.99) (*n *= 77)	6.65 (5.73, 7.72) (*n *= 73)
Week 18	Mean (95% CI)	7.89 (6.96, 8.95) (*n *= 102)	8.01 (6.77, 9.48) (*n *= 77)	8.27 (7.06, 9.69) (*n *= 75)	6.91 (5.90, 8.10) (*n *= 74)	7.04 (6.07, 8.15) (*n *= 74)
Week 24	Mean (95% CI)	8.12 (7.23, 9.13) (*n *= 102)	8.52 (7.30, 9.95) (*n *= 77)	8.15 (7.02, 9.46) (*n *= 74)	7.69 (6.60, 8.96) (*n *= 76)	7.62 (6.66, 8.73) (*n *= 74)
Week 13 vs. Week 12	Percent change (95% CI)^*a*^	–2.4 (–6.7, 2.0) (*n *= 101)	–4.0 (–8.8, 1.1) (*n *= 77)	0.1 (–5.3, 5.7) (*n *= 75)	–4.7 (–9.1, –0.1) (*n *= 77)	–5.0 (–9.6, –0.1) (*n *= 72)
Week 18 vs. Week 12	Percent change (95% CI)	4.3 (–2.9, 12.1) (*n *= 101)	–6.4 (–13.8, 1.5) (*n *= 77)	–0.5 (–9.2, 9.0) (*n *= 75)	–5.8 (–14.5, 3.7) (*n *= 74)	0.8 (–7.9, 10.4) (*n *= 73)
Week 24 vs. Week 12	Percent change (95% CI)	7.8 (0.0, 16.2) (*n *= 101)	–0.4 (–10.9, 11.3) (*n *= 77)	–0.8 (–12.5, 12.4) (*n *= 74)	3.8 (–5.8, 14.5) (*n *= 76)	9.1 (–2.3, 21.7) (*n *= 73)
^***a***^Percent change = (geometric mean ratio – 1) × 100.						

*Description of treatment effects in the second phase.* Table 3 presents geometric means of bAs, and the percent change in bAs from week 12 to a follow-up time, along with 95% CIs, in the second phase of the study. Mean ln-bAs at week 12 did not differ significantly between the FA400/FA and FA400/placebo groups (*p* = 0.73) or between the FA800/FA and FA800/placebo groups (*p* = 0.53). The percent change in geometric mean of bAs since week 12 did not differ significantly between the groups with and without continuation of FA supplements (i.e., FA400/FA vs. FA400/placebo and FA800/FA vs. FA800/placebo). Likewise, the percent change in geometric mean of urinary As (micrograms per gram creatinine) since week 12 did not differ significantly between the FA400/FA and FA400/placebo groups, and the FA800/FA and FA800/placebo groups (see Supplemental Material, Table S2). These patterns suggest that there was no rebound in bAs related to cessation of FA supplementation. Because the FA800/FA and FA800/placebo groups were the only groups to maintain a lower geometric mean bAs than the placebo group at every follow-up time point, and because there was no evidence of a rebound in bAs related to cessation of FA supplementation, we conducted a secondary analysis in which we combined the FA800/FA and FA800/placebo groups in the second phase of the study. Overall, the combined FA800 groups had a greater within-person decline in bAs since baseline (–12.3%; 95% CI: –19.6%, –4.3%) than the placebo group (–2.3%; 95% CI: –8.6%, 4.4%) at the end of the 24 weeks (*p* = 0.05).

**Figure 4:**
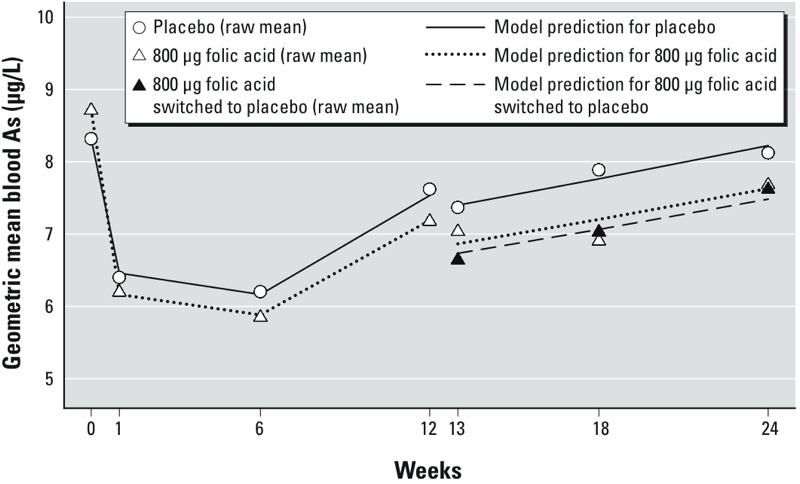
Plot of geometric mean blood arsenic (μg/L) in the placebo group and 800-μg folic acid group by week of treatment. Symbols represent raw means, and lines connect the predicted means from a linear model with repeated measures of ln-blood As.

*Modeling the treatment effect in the FA800 group.* We observed that the geometric means of bAs after baseline in the FA800 group were consistently lower than in the placebo group, with a parallel pattern of trend (Figure 4). We applied linear models with repeated measures of ln-bAs using all time points in order to evaluate the treatment effect of FA800 over 24 weeks. The estimated parameters of the initial model with categorical variables for time, treatment group, and time by treatment interaction, suggested a model simplification using fewer main effect parameters to describe the time trend of ln-bAs in the placebo group, and using fewer interaction terms for the treatment group differences in the time trend. The geometric means of bAs over time were all close to the model-based estimates, suggesting that the model fit the data well (Figure 4). Given the model parameters, we estimated the group mean difference in ln-bAs change since baseline in the first phase (difference = –0.09, 95% CI: –0.18, –0.01, *p* = 0.03) (Table 4), which indicated that the average decline in ln-bAs from baseline in the FA800 group was greater than in the placebo group. In the second phase, compared to the change in ln-bAs since baseline in the placebo group, the two FA800 groups had significantly greater reductions in ln-bAs since baseline (FA800/FA: difference = –0.12, 95% CI: –0.24, –0.00, *p* = 0.04; FA800/placebo: difference = –0.14, 95% CI: –0.26, –0.02, *p* = 0.02). The small difference in the change in ln-bAs since baseline between the FA800/FA group and the FA800/placebo group was not statistically significant (difference = 0.02, 95% CI: –0.09, 0.12, *p* = 0.72), indicating a lack of bAs rebound in the FA800/placebo group.

**Table 4:** Group mean difference in ln-blood As decline since baseline estimated from repeated measures model.*^a^*

Time period	Group comparison	Group mean difference in ln-bAs decline from baseline (95% CI)	*p*-Value
Week 1–12	FA800 vs. placebo	–0.09 (–0.18, –0.01)	0.03
Week 13–24	FA800/placebo vs. placebo	–0.14 (–0.26, –0.02)	0.02
	FA800/FA vs. placebo	–0.12 (–0.24, –0.00)	0.04
	FA800/FA vs. FA800/placebo	0.02 (–0.09, 0.12)	0.72
^***a***^The model parameters: six for the time trend in the placebo group, one for the difference between FA800 and placebo groups, and three for the time by treatment group interactions to describe the differences in the time trend between the FA800 group(s) and the placebo group. No other covariates were adjusted for in this model.			

## Discussion

Supplementation of 800 μg FA/day to a study population comprised of both folate-deficient and -sufficient individuals lowered bAs to a greater extent than placebo over the course of this 24-week randomized clinical trial. This result is in accordance with our previous clinical trial in folate-deficient adults, in which As-removal filters were not provided and supplementation with 400 μg FA lowered bAs to a greater extent than placebo over 12 weeks (Gamble et al. 2007). The enhanced decline in bAs related to 800-μg FA supplementation was observed as early as 1 week after supplementation began, which is also in accordance with the enhanced As methylation observed after 1 week of supplementation with 400 μg FA in our previous clinical trial in folate-deficient adults (Gamble et al. 2006). Additionally, the magnitude of the reduction in bAs (mean within-person decline of 12%) is similar to that observed in our previous clinical trial (14%). Synthesis of SAM, the methyl donor for As methylation, relies on folate-dependent one-carbon metabolism (Figure 1). By increasing methylation of InAs to DMA, folate facilitates the elimination of As in urine, resulting in a lowering of bAs (Gamble et al. 2007). The finding that FA has a beneficial effect in the general Bangladeshi population even beyond the effect of As remediation alone has great public health significance. For example, on the basis of a prospective study of bAs and incident skin lesions in Bangladesh (Hall et al. 2006), we have calculated that a 12% decline in bAs (as observed in the FA800 group) would be associated with an 8.2% decline (95% CI: 5.8%, 10.5%) in the incidence rate of skin lesions.

We did not observe that cessation of FA supplementation at week 12 was associated with a rebound in bAs, compared with remaining on FA supplementation. This indicates that the decline in bAs achieved with 12 weeks of supplementation with FA800 was sustained up to 12 weeks after supplementation had ceased. Although plasma folate returned to near baseline levels by week 24 in the FA800/placebo group, RBC folate remained elevated substantially above baseline levels. Plasma folate is highly responsive to recent intake of folate, whereas RBC folate is more indicative of long-term folate status and tissue folate stores (Institute of Medicine 1998). Because the liver is the primary site of As metabolism (Drobná et al. 2010), it is possible that liver folate, like RBC folate, was sustained after cessation of FA supplementation, resulting in a sustained lowering of bAs in the FA800/placebo group. Alternatively, we may not have been able to detect a rebound in bAs after week 12 related to cessation of FA800 because of the increase in bAs after week 6 in all treatment groups (discussed in further detail below).

FA400, creatine, and creatine+FA400 were not effective in lowering bAs to a greater extent than the placebo group over the course of the trial. The reason that FA400 did not lower bAs in this study may be an issue of insufficient dose, because we did observe that FA800 decreased bAs from baseline to a greater extent than placebo. Plots of bAs over time stratified by folate deficiency (plasma folate < 9 nmol/L) suggest that FA400 may have been effective in lowering bAs in folate-deficient individuals (data not shown), as we have observed in our previous trial in folate-deficient adults (Gamble et al. 2007). This was solely an exploratory analysis, because the sample sizes in the folate-deficient subgroups were small (placebo *n* = 22, FA400 *n* = 36, FA800 *n* = 27), and we did not have adequate statistical power to perform stratified analyses.

Because of the strong positive association between uCrn and As methylation observed in many of our studies and those of others, and the knowledge that supplemental creatine downregulates creatine biosynthesis in rodents (Stead et al. 2001), we had hypothesized that creatine supplementation would “spare” SAM and thereby enhance As methylation and lower bAs. However, we did not observe that supplementation with 3 g creatine/day lowered bAs to a greater extent than placebo. It seems unlikely that this may also be an issue of insufficient dose, because we observed a significant decrease in plasma GAA in the creatine groups, indicating downregulation of endogenous creatine synthesis. However, the functional effect of this down-regulation may be smaller than anticipated due to long-range allosteric interactions that tightly regulate hepatic SAM concentrations (Wagner et al. 1985).

FA supplementation is a very promising nutritional intervention for lowering bAs; however, there may yet be “room for improvement” in enhancing As methylation, because AS3MT activity is unlikely to reach a plateau at physiological SAM concentrations [based on *in vitro* AS3MT activity (Song et al. 2010) and liver SAM concentrations in rats (Finkelstein et al. 1982)]. Because FA is not a methyl donor per se, but rather recruits a methyl group to serve as a cofactor for remethylation of homocysteine to methionine, FA cannot increase SAM if methyl groups are limiting (Benevenga 2007). Future studies should examine whether supplementation with 5-methyltetrahydrofolate, or co-supplementation with methyl donors such as choline or betaine, can provide an additional benefit in lowering bAs.

All treatment groups experienced a rebound in mean bAs after week 6. This was unexpected, given that As exposure had ceased at the beginning of the study with the use of water filters. The most likely explanation for this occurrence is that there was a decline in compliance of filter usage. A survey administered to the study participants approximately 1.5 years after the end of the clinical trial revealed that most (95%) had stopped using their filters by then because the filter flow rates had declined. Additionally, in an 11-week pilot study of *n* = 12 As-exposed Bangladeshi adults drinking bottled water supplied by our field staff, a rebound in bAs was not observed after the initial decline in bAs, further suggesting an issue with filter compliance in the current study. Alternatively, we cannot rule out other possibilities such as release of As from tissue stores (e.g., bone) following reduction in As exposure, given that there is some evidence that As may influence bone turnover (Hu et al. 2012; Wu et al. 2014). Although the explanation for the rebound in bAs is not clear at this time, our findings do suggest that provision of costly individual household-use water filters does not represent a feasible long term solution for the As problem in a real-world setting.

## Conclusions

Although removing exposure to As is the primary prevention approach for As-induced diseases, massive exposure reduction is complicated by the high cost of water filters and their monitoring and maintenance, the limited effectiveness of water filters in real-world settings, and the limited availability of low As wells in some regions. Additionally, long-term chronic exposure to As may lead to an increased body burden of As that persists long after exposure is removed (National Research Council 2013). Nutritional supplementation presents a low-risk and low-cost preventative treatment option for populations with past exposure who are still at risk for As-induced disease (Smith et al. 2012), and perhaps for populations currently exposed to As. We have demonstrated that even with provision of As-removal water filters, supplementation with 800 μg FA lowered bAs to a greater extent than placebo. Future studies should examine the impact of folate supplementation on risk for As-induced diseases. Although at least 57 countries worldwide currently have mandatory folate fortification in place in accordance with World Health Organization guidelines (Zimmerman 2011), those countries with the most significant problems of As-contaminated drinking water, including Bangladesh, India, Cambodia, and Pakistan, are not among them. As has been demonstrated in Western countries such as the United States and Canada, folate fortification can nearly eradicate folate deficiency and its associated consequences; in As-endemic countries it may have the additional benefit of facilitating a partial reduction in the extensive public health burden of As exposure.

## Supplemental Material

(166 KB) PDF
