# Supplementary material for: Folic Acid and Creatine as Therapeutic Approaches to Lower Blood Arsenic: A Randomized Controlled Trial
Source: Environ Health Perspect. 2015 May 15;123(12):1294–301. doi: 10.1289/ehp.1409396 (PMC4671237; doi:10.1289/ehp.1409396)
Supplement: (166 KB) PDF [file ehp.1409396.s001.acco.pdf]

*Environ Health Perspect*
doi: 10.1289/ehp.1409396

**Note to Readers:** *EHP* strives to ensure that all journal content is accessible to all readers. However, some figures and Supplemental Material published in *EHP* articles may not conform to 508 standards due to the complexity of the information being presented. If you need assistance accessing journal content, please contact ehp508@niehs.nih.gov. Our staff will work with you to assess and meet your accessibility needs within 3 working days.

# Supplemental Material

# Folic Acid and Creatine as Therapeutic Approaches to Lower Blood Arsenic: A Randomized Controlled Trial

Brandilyn A. Peters, Megan N. Hall, Xinhua Liu, Faruque Parvez, Tiffany R. Sanchez, Alexander van Geen, Jacob L. Mey, Abu B. Siddique, Hasan Shahriar, Mohammad Nasir Uddin, Tariqul Islam, Olgica Balac, Vesna Ilievski, Pam Factor-Litvak, Joseph H. Graziano, and Mary V. Gamble

### Table of Contents

**Table S1**. Geometric mean (95% CI) of urinary arsenic (µg/g creatinine) and the percent change in urinary arsenic from baseline to week 12 by First Phase treatment group.

**Table S2**. Geometric mean (95% CI) of urinary arsenic (µg/g creatinine) and the percent change in urinary arsenic from week 12 to week 24 by Second Phase treatment group.

**Table S1**. Geometric mean (95% CI) of urinary arsenic (µg/g creatinine) and the percent change in urinary arsenic from baseline to week 12 by First Phase treatment group.

| Time or time comparison | Statistic | Placebo | FA400µg | FA800µg | Creatine | Creatine + FA400µg |
|---|---|---|---|---|---|---|
| **Baseline** | Mean (95% CI) | 253.0 (224.5, 285.1) (N=102) | 257.0 (229.5, 287.7) (N=153) | 259.7 (235.9, 285.9) (N=151) | 263.6 (231.8, 299.8) (N=101) | 272.0 (244.6, 302.5) (N=103) |
| **Week 1** | Mean (95% CI) | 191.3 (170.7, 214.4) (N=102) | 198.8 (178.4, 221.6) (N=151) | 183.5 (168.9, 199.4) (N=150) | 176.4 (157.2, 197.8) (N=101) | 180.8 (161.5, 202.5) (N=103) |
| **Week 6** | Mean (95% CI) | 191.7 (169.2, 217.2) (N=102) | 201.4 (177.6, 228.5) (N=152) | 178.6 (162.6, 196.3) (N=149) | 173.3 (152.0, 197.7) (N=100) | 177.4 (155.0, 202.9) (N=103) |
| **Week 12** | Mean (95% CI) | 208.0 (181.3, 238.8) (N=102) | 225.8 (200.6, 254) (N=150) | 191.9 (173.1, 212.6) (N=148) | 195.6 (171.2, 223.4) (N=99) | 195.1 (169.1, 225.2) (N=102) |
| **Week 1 vs. Baseline** | Percent change (95% CI)[a] | -24.4 (-31.2, -16.9) (N=102) | -22.9 (-28.1, -17.4) (N=151) | -29.3 (-34.2, -24.1) (N=150) | -33.1 (-37.8, -28.0) (N=101) | -33.5 (-38.3, -28.3) (N=103) |
| **Week 6 vs. Baseline** | Percent change (95% CI) | -24.2 (-31.4, -16.3) (N=102) | -22.2 (-28.6, -15.3) (N=152) | -31.1 (-37.3, -24.3) (N=149) | -33.9 (-40.6, -26.4) (N=100) | -34.8 (-42.0, -26.7) (N=103) |
| **Week 12 vs. Baseline** | Percent change (95% CI) | -17.8 (-26.0, -8.7) (N=102) | -13.1 (-20.0, -5.6) (N=150) | -25.9 (-33.1, -18.0) (N=148) | -25.9 (-32.9, -18.1) (N=99) | -28.5 (-36.3, -19.8) (N=102) |

[a]Percent change = (geometric mean ratio – 1)*100.

**Table S2**. Geometric mean (95% CI) of urinary arsenic (µg/g creatinine) and the percent change in urinary arsenic from week 12 to week 24 by Second Phase treatment group.

| Time or time comparison | Statistic | Placebo | FA400µg continued | FA400µg switched to placebo | FA800µg continued | FA800µg switched to placebo |
|---|---|---|---|---|---|---|
| **Week 12** | Mean (95% CI) | 208.0 (181.3, 238.8) (N=102) | 231.1 (195.9, 272.7) (N=77) | 220.2 (185.4, 261.6) (N=73) | 199.4 (173.3, 229.6) (N=75) | 184.4 (158.3, 214.8) (N=73) |
| **Week 13** | Mean (95% CI) | 216.8 (188.7, 249.1) (N=102) | 228.4 (190.0, 274.7) (N=77) | 230.3 (192.7, 275.2) (N=75) | 206.1 (180.5, 235.2) (N=76) | 178.1 (151.7, 209.0) (N=71) |
| **Week 18** | Mean (95% CI) | 242.9 (215.4, 273.9) (N=102) | 228.4 (191.3, 272.6) (N=77) | 236.1 (198.5, 280.8) (N=75) | 205.2 (175.8, 239.4) (N=74) | 191.9 (164.8, 223.5) (N=72) |
| **Week 24** | Mean (95% CI) | 228.7 (202.1, 258.9) (N=100) | 238.0 (199.9, 283.4) (N=77) | 226.7 (191.6, 268.3) (N=73) | 221.8 (189.4, 259.6) (N=73) | 207.5 (176.0, 244.6) (N=71) |
| **Week 13 vs. Week 12** | Percent change (95% CI)[a] | 4.2 (-3.6, 12.6) (N=102) | -1.2 (-9.5, 7.9) (N=77) | 4.9 (-3.3, 13.7) (N=73) | 2.2 (-5.5, 10.6) (N=75) | -4.1 (-11.1, 3.4) (N=71) |
| **Week 18 vs. Week 12** | Percent change (95% CI) | 16.8 (6.1, 28.5) (N=102) | -1.2 (-11.4, 10.2) (N=77) | 9.3 (-2.1, 22.1) (N=72) | 2.8 (-8.3, 15.3) (N=73) | 3.7 (-6.1, 14.4) (N=72) |
| **Week 24 vs. Week 12** | Percent change (95% CI) | 10.1 (-0.0, 21.1) (N=100) | 3.0 (-7.7, 14.9) (N=77) | 6.1 (-6.6, 20.6) (N=71) | 12.8 (-0.7, 28.1) (N=73) | 11.0 (-0.5, 23.8) (N=71) |

[a]Percent change = (geometric mean ratio – 1)*100.
